# COVID-19’s Influence on Information and Communication Technologies in Long-Term Care: Results From a Web-Based Survey With Long-Term Care Administrators

**DOI:** 10.2196/32442

**Published:** 2022-01-12

**Authors:** Amy M Schuster, Shelia R Cotten

**Affiliations:** 1 Department of Sociology, Anthropology, and Criminal Justice Clemson University Clemson, SC United States

**Keywords:** COVID-19, pandemic, socioemotional needs, long-term care, nursing home facility, assisted living facility, elderly, older adults, information and communication technologies, support, emotion, needs, access, connection, communication, engagement

## Abstract

**Background:**

The prevalence of COVID-19 in the United States led to mandated lockdowns for long-term care (LTC) facilities, resulting in loss of in-person contact with social ties for LTC residents. Though information and communication technologies (ICTs) can be used by LTC residents to support their socioemotional needs, residents must have access to ICTs to use them.

**Objective:**

This study explored ICT access and use in LTC facilities and how LTC facilities adapted to try to enhance social connections for their residents during the COVID-19 pandemic.

**Methods:**

LTC administrators in South Carolina (United States) were invited to complete a web-based survey exploring ICT access and use in LTC facilities and whether access and use changed as a result of the COVID-19 pandemic.

**Results:**

LTC administrators (N=70, 12 nursing homes [NHs], and 58 assisted living facilities [ALFs]) completed the web-based survey. Since March 2020, a total of 53% (37/70) of the LTC facilities have purchased ICTs for residents’ use. ICTs have mainly been used for videoconferencing with family members (31/36, 86%), friends (25/36, 69%), and health care providers (26/36, 72%). NHs were 10.23 times more likely to purchase ICTs for residents’ use during the COVID-19 pandemic than ALFs (odds ratio 11.23, 95% CI 1.12-113.02; *P*=.04). Benefits of ICT use included residents feeling connected to their family members, friends, and other residents. Barriers to ICT use included staff not having time to assist residents with using the technology, nonfunctional technology, and residents who do not want to share technology.

**Conclusions:**

Our results suggest that over half of the LTC facilities in this study were able to acquire ICTs for their residents to use during the COVID-19 pandemic. Additional research is needed to explore how residents adapted to using the ICTs and whether LTC facilities developed and adopted technology integration plans, which could help them be prepared for future situations that may affect LTC residents’ engagement and communication opportunities, such as another pandemic.

## Introduction

As a result of the COVID-19 pandemic, nursing homes (NHs), assisted living facilities (ALFs), and other long-term care (LTC) facilities were required to restrict access to the public since March 2020 [1]. Many of these communities were not prepared to operate in this type of emergency as they were faced with personal protective equipment and staff shortages [2]. LTC facilities were even less prepared for the increased socioemotional needs, which arose for residents due to the loss of in-person contact with family and friends because of the mandated facility lockdown [3-5].

Loneliness and social isolation have long been salient issues for LTC facilities [6,7]. Change in residence, the death of family members and friends, and increased medical needs have been associated with increased loneliness and isolation among LTC residents [6,8-13]. As a way to address residents’ quality of life, LTC facilities abide by federal regulations to facilitate resident communication opportunities with people within and outside of the facility. For example, nursing homes are federally mandated to provide telephone access for each resident and are required to provide internet access if it is available at the facility [14]. In addition to following federal regulations, some LTC facilities have also started to incorporate information and communication technologies (ICTs) for residents’ use in the facility [15-17]. ICTs are devices and applications that provide the potential for unlimited connectivity and communication through technology such as desktop computers, laptops, tablets, smartphones, the internet, social media platforms, and videoconferencing [16,18]. Some segment of LTC residents can use ICTs, although residents’ age, education level, interest in technology, and level of care may influence ICT use (Seifert A and Cotton S, unpublished data, 2021) [19,20].

The advent of the COVID-19 pandemic and the mandated LTC lockdowns necessitated that LTC facilities determine ways to help residents stay engaged with their social ties [21] and continue to receive medical care [22-24]. Most studies examining how the COVID-19 pandemic has affected the lives of older adults have concentrated on community-dwelling adults. Among the COVID-19–related studies on LTC [25]**,** the main focus has been on the medical circumstances (eg, cases, outbreaks, and personal protective equipment) pertaining COVID-19 [2,26-41], LTC employees [2,42-47], communication with family members [36,48], or provision of training on LTC issues related to the COVID-19 pandemic [24,49,50].

Among studies focusing on ICT access and use by LTC residents since the onset of the COVID-19 pandemic, almost none have examined ICT changes that facilities have implemented to address the potential socioemotional impacts on residents. ICTs have been used for telehealth purposes in medical screening and health care management of LTC residents [48,51-53]. A few studies have examined how LTC residents used ICTs for social purposes to communicate with those outside of the LTC facility. Telephone calls were reported by family members of LTC residents as the primary method of communication with LTC residents [54,55], followed by texting [54] and videoconferencing [54,55] during the mandatory lockdown. However, LTC residents reported that they preferred videoconferencing with family members rather than telephone calls [56]. Telephone calls were also employed in outreach interventions targeting LTC residents at risk for social isolation during the COVID-19 pandemic [57,58].

Three studies have assessed ICT availability in LTC facilities and use by LTC residents during the COVID-19 pandemic [3,48,59]. Montgomery et al [3] examined ICT use among a sample of 365 nursing home residents and found that 40% of the respondents owned a device, 47% indicated that their nursing home had computers or tablet devices, and 67% said that their nursing home offered free unlimited access to the internet via Wi-Fi. Ickert et al [59] evaluated the ICT resources in 10 care homes in Canada and found that all 10 care homes had tablet devices available for use. However, barriers to use existed, including the following: (1) age of the tablets, which could prevent videoconferencing apps from updating, or image disturbances during videoconferencing and (2) weak Wi-Fi infrastructure resulting in all videoconferencing having to occur in one area of the care home. Staff members were the critical link in facilitating video communication between residents and their family members. Staff members scheduled the videoconferencing visits, helped residents to the area in the home where they could participate in the videoconference call, assisted residents in using the tablets, and cleaned the tablet devices after each use. Marin et al [48] surveyed a staff member at each of the 46 ALFs in Rhode Island, which received donated tablets. Of the 46 ALFs, 11 of the staff members completed a web-based survey 2 weeks after the tablets were distributed. Survey responses indicated that the tablets were predominately used by residents to video chat with their family members (90.9%).

Though these 3 studies provide some insights into availability and use of ICTs in LTC facilities, they do not offer insights into how LTC facilities adapted during the pandemic to lessen potential social isolation and loneliness among their residents. To address this deficiency, this study explored (1) how LTC institutions modified technology access in their facilities and (2) the challenges that arose with these adaptations.

## Methods

### Recruitment and Data Collection

At the onset of the COVID-19 pandemic, the state of South Carolina placed contact limitations for residents of NHs and ALFs (known as community residential care facilities in South Carolina) [60]. Hence, we included both NHs and ALFs in this study as they experienced the same government-mandated restrictions.

LTC administrators in South Carolina were invited to complete a web-based survey from November to December 2020 to explore ICT access and use in LTC facilities and whether access and use changed as a result of the COVID-19 pandemic. Email contact information for 193 NH and 496 ALF administrators was obtained from the South Carolina Department of Health and Environmental Control (DHEC) website [61]. LTC administrators were recruited to participate in the Qualtrics survey through email, which included a secure weblink to access the survey. After sending the initial email request for participation, follow-up emails were sent at 3 days and 13 days. The Qualtrics survey was composed of 20 pages with 1-3 questions per page, there were adaptive questions based on the response to other items, and the survey took approximately 20 minutes to complete. During the survey, participants were able to review and change their answers using a back button. In total, 70 LTC administrators (12 NHs and 58 ALFs; 1 participant per site) completed the Qualtrics survey. Informed consent, which included the estimated time to complete the survey, data protection, the purpose of the study, and the principal investigator, was reviewed prior to the start of the survey. Participation was voluntary, and LTC administrators who chose to participate in the study clicked “yes” in agreement and began the survey. No incentives were offered for participation. This study was reviewed and approved by the university institutional review board.

### Measures

#### Facility Characteristics

Participants were first asked general information about their LTC facility. The type of LTC facility was determined by the name of the facility, “What is the name of your facility?” and which type of LTC facility the name was associated with on the DHEC website [61]. Where the facility was located in South Carolina was assessed by an open-ended question, “In which city is your facility located?” The number of employees was measured with 2 questions: “How many full-time employees does your facility have?” and “How many part-time employees does your facility have?” Response options for both questions ranged from 1 to 100 in intervals of 1 with the final response option of “more than one-hundred.” The bed count was assessed numerically with the question, “How many beds does your facility have?” and then converted into size groups (>50 beds, 51-149 beds, 150 or more beds) following standard categorization [62]. Bed occupancy was measured by two questions: “What percentage of beds was occupied in February 2020, prior to COVID-19, in your facility?” and “What is the percentage of beds occupied now in your facility?” For both questions, response options ranged from 5 to 100 in intervals of 5. Facility ownership was assessed by the measure, “What is the ownership type of your facility?” with 3 response options (for profit, nonprofit, and federal or state).

#### ICT Access and Use

Facility technology preparedness was measured with the question, “How technologically prepared was your organization to address the social distancing need for residents as a result of COVID19?” Response options included the following: “Fully prepared,” “Mostly prepared,” “Somewhat prepared,” and “Not prepared.” We then assessed the facility technology capabilities with response options of “Yes,” “No,” or “Do not know”, to the following questions: “Does your facility have internet access?” “Does your facility have WiFi?” “Are residents able to access the internet?” “Are residents able to access WiFi?” and “Does your facility have a dedicated employee who helps residents with technology needs/issues?” Technology provided by the facility for residents’ use, prior to the COVID-19 pandemic, was assessed with one question, “Prior to February 2020, which type of technology did your facility provide for residents’ use?” Response options included the following: “TV,” “Radio,” “Desktop computers,” “Laptops,” “Smartphones,” and “Tablets”; respondents could select all that applied. Residents’ technology use was measured with a “Yes” or “No” response to “My residents use these technologies: Laptops, tablets, and smartphones.” The participants who responded “No” were then prompted with the follow-up question, “Why do you think that residents in your facility do not use laptops/tablets/smartphones?” Response options included, selecting all that apply, the following: “Do not have a need,” “Poor WiFi/bandwidth capability/capacity,” “Physical infrastructure of building,” “Cost is prohibitive,” or “Other (please specify).”

#### Changes in Facility ICTs, Access, and Use Since the Onset of the COVID-19 Pandemic

Next, we asked technology-related questions about use in the LTC facility since the COVID-19 pandemic. Technology spending was assessed through four questions: (1) “How much did your organization adjust its technology spending for residents due to COVID-19?” with response options including “Increased spending by more than 50%,” “Increased spending by 25-50%,” “Increased spending less than 25%,” “No change,” and “Decreased spending”; (2) “Since February 2020, has your facility purchased new technology for residents’ use?” with response options including “Yes” and “No.” The participants who responded with “Yes” were then prompted with four follow-up questions: “Which technology has been purchased for residents’ use?” with response options including “Laptops,” “Tablets,” “Cellphones,” “Smartphones,” or “Other (please specify)”; and “What type(s) of funds were used to purchase these devices?” with response options including (select all that apply) “Donation,” “the CMS COVID-19 Communicative Technology grant,” “Facility funds,” or “Other (please specify).” An open-ended question was asked: “Why was this new technology purchased?” Lastly, we asked, “How did residents learn to use this technology?” with response options including “Staff member helped them learn,” “Learned on their own,” “Another resident helped them learn,” and “Other (please specify).” New technology used by residents was assessed through three questions: (1) “How has this technology provided by your organization been used by residents?” with response options including (select all that apply) “Playing games,” “Video conferencing,” “Email,” “Searching for information,” “Shopping,” and “Other (please specify).” The number of residents using the technology was measured by two questions: “What percentage of residents have used this technology?” and “What percentage of residents have been unable to use the technology provided by your organization due to health or other impairments?” with response options for both questions ranging from 5 to 100, in intervals of 5.

#### Benefits and Barriers to ICT Use

Finally, resident changes since using technology were measured with two questions including (1) “Have there been any positive changes since residents started to use the new technology?” with response options including (select all that apply) “Decreased negative behaviors from residents,” “Residents socializing more,” “Residents feel connected to family members,” “Residents feel connected to friends,” “Family members feel connected to other residents,” and “Other (please specify)” and (2) “Have there been any negative changes since residents started to use the new technology?” with response options including (select all that apply) “Staff don’t have time to assist residents with technology,” “Broken technology,” “Stolen technology,” “Infection spread due to sharing technology,” “Residents do not want to share technology,” and “Other (please specify).”

### Analysis

Questionnaires that had been completed up to 73% or more were included in the analysis. Given the exploratory nature of this study and the small sample size, the data were initially analyzed descriptively. A binary logistic regression model was used to investigate whether facility characteristics (ie, type, ownership, and bed size) influenced ICTs purchased during the COVID-19 pandemic. In line with the aim of this study, exploring ICT changes in LTC facilities during the pandemic, the dependent variable was the binary measure that assessed whether facilities purchased ICTs for residents’ use during the COVID-19 pandemic.

## Results

### Facility Characteristics

**Figure 1:**
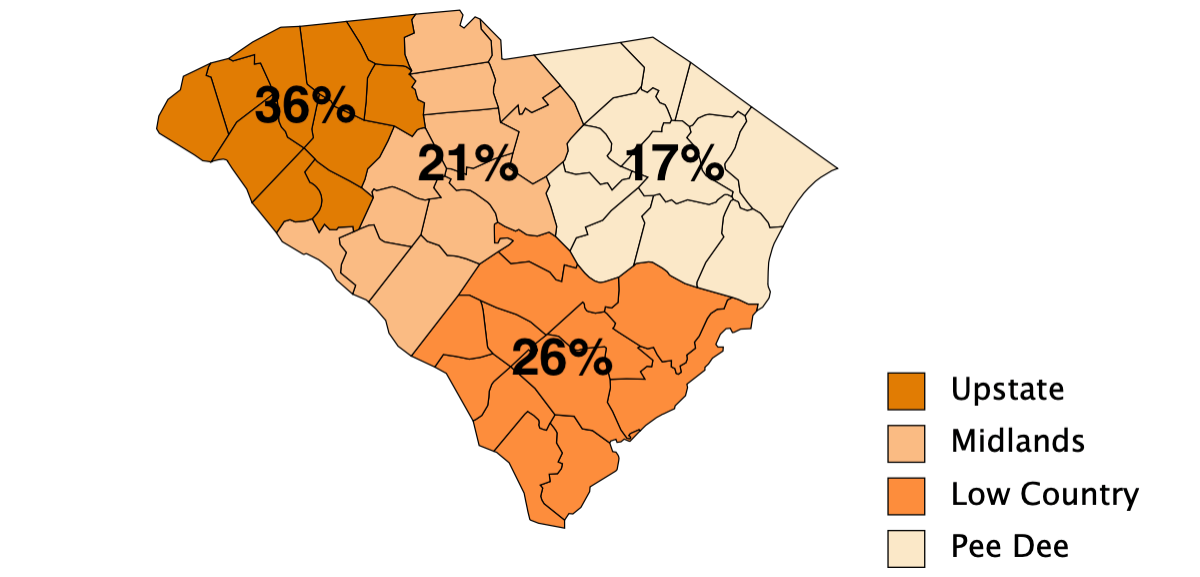
Proportion of long-term care facilities participating in this study by regions in South Carolina, United States.

The LTC facilities (N=70) were located throughout South Carolina in the Upstate (25/70, 36%), Low Country (18/70, 26%), Midlands (15/70, 21%), and Pee Dee regions (12/70, 17%) (Table 1 and Figure 1). The majority of the facilities were for-profit ownership (54/70, 77%). In total, 58 of the facility administrators that responded were from ALFs, with the remaining 12 administrators being from NHs. In total, 44% (31/70) of the facilities had a medium bed size (26-100 beds). Half of the ALFs (29/58) had 25-100 beds, while 83% (10/12) of the NHs had greater than 100 beds. Prior to the COVID-19 pandemic, administrators reported that, on average, 82% (SD 24.4%) of the beds were occupied (Table 2). Since the COVID-19 pandemic, administrators reported, on average, 74% (SD 23.4%) of the beds have been occupied. The facilities had, on average, 37 full-time employees (SD 35.6) and 14 part-time employees (SD 31.1). Most of the administrators (37/70, 57%) thought that their facility was at least mostly technologically prepared to address the social distancing needs for their residents that arose as a result of the COVID-19 pandemic.

**Table 1:** Facility characteristics.

Characteristics		Total (n=70), n (%)	Assisted living facilities (n=58), n (%)	Nursing homes (n=12), n (%)
**Region in South Carolina**				
	Low Country	18 (26)	17 (29)	1 (9)
	Midlands	15 (21)	12 (21)	3 (25)
	Pee Dee	12 (17)	8 (14)	4 (33)
	Upstate	25 (36)	21 (36)	4 (33)
**Ownership type**				
	For profit	54 (77)	44 (76)	10 (83)
	Nonprofit	13 (19)	11 (19)	2 (17)
	Federal or state	3 (4)	3 (5)	0
**Bed size**				
	Small (fewer than 25 beds)	22 (31)	22 (38)	0
	Medium (26-100 beds)	31 (44)	29 (50)	2 (17)
	Large (101 or more beds)	17 (25)	7 (12)	10 (83)
**Facility technology preparedness^a^**				
	Fully prepared	13 (20)	11 (20)	2 (20)
	Mostly prepared	24 (37)	21 (39)	3 (30)
	Somewhat prepared	23 (35)	18 (33)	5 (50)
	Not prepared	5 (8)	5 (9)	0

^a^Missing data from 3 assisted living facilities and 2 nursing homes.

**Table 2:** Additional facility characteristics.

Characteristics		Score, mean (SD); range
**Percent of beds occupied prior to COVID-19**		
	Total	82.21 (24.4); 5 to 100
	Assisted living facilities	80.34 (26.4); 5 to 100
	Nursing homes	91.25 (4.8); 80 to 95
**Percent of beds occupied during COVID-19**		
	Total	73.93 (23.4); 5 to 100
	Assisted living facilities	73.73 (25.5); 5 to 100
	Nursing homes	75.83 (8.2); 60 to 90
**Full-time employees**		
	Total	37.5 (35.6); 1 to ≥101
	Assisted living facilities	25.6 (26.1); 1 to ≥101
	Nursing homes	95 (9.6); 75 to ≥101
**Part-time employees^a^**		
	Total^a^	14.5 (19.1); 1 to 76
	Assisted living facilities^a^	11.7 (29.6); 1 to ≥101
	Nursing homes	44.2 (22); 15 to 76

^a^Missing responses from 7 assisted living facilities.

### ICT Access and Use

Most of the facilities provided internet (69/70, 99%) and Wi-Fi (66/70, 94%) access, although not all of them allowed residents to access the internet (59/69, 86% compared to 99% who had internet access) and Wi-Fi (57/66, 86% compared to 94% who had Wi-Fi access). Prior to February 2020, the top 2 ICTs provided by LTC facilities for residents’ use were televisions (65/67, 97%) and radios (53/67, 79%; Table 3). In total, 33 of the 70 (47%) facilities have a dedicated employee to provide ICT support to residents. In total, 59% (38/64) of LTC administrators reported that their residents used laptops, 78% (50/64) of LTC administrators reported that their residents used tablet devices, and 96% (61/64) of LTC administrators reported that their residents used smartphones. Of the LTC administrators who reported that their residents did not use laptops, tablet devices, or smartphones, the predominant reasons for nonuse were lack of need (18/35, 51%) or prohibitive cost (7/35, 20%).

**Table 3:** Access to and use of information and communication technologies.

Technologies			Total (N=70), n (%)		Assisted living facilities (n=58), n (%)		Nursing homes (n=12), n (%)
**Internet or Wi-Fi**							
	Facility has internet access	69 (99)		57 (98)		12 (100)	
	Residents able to access the internet	59 (86)		47 (82)		12 (100)	
	Facility has Wi-Fi	66 (94)		54 (93)		12 (100)	
	Residents able to access the Wi-Fi	57 (86)		46 (85)		11 (92)	
**Information and communication technologies available for residents to use^a^**							
	Television	65 (97)		55 (98)		10 (91)	
	Radio	53 (79)		47 (84)		6 (55)	
	Tablet device	24 (36)		19 (34)		5 (45)	
	Smartphone	20 (30)		17 (30)		3 (27)	
	Desktop computer	20 (30)		15 (27)		5 (45)	
	Laptop	17 (25)		12 (21)		5 (45)	
Dedicated employee to help residents with information and communication technologies			33 (47)		27 (48)		6 (50)
**Information and communication technologies that residents use^b^**							
	Smartphones	61 (96)		49 (94)		12 (100)	
	Tablet devices	50 (78)		38 (73)		12 (100)	
	Laptops	38 (59)		27 (52)		11 (92)	
**Reason why residents do not use information and communication technologies**							
	Do not have a need	18 (51)		17 (50)		1 (100)	
	Cost is prohibitive	7 (20)		7 (21)		0	
	Poor Wi-Fi, bandwidth capability, or capacity	6 (2)		2 (6)		0	
	Other (cognitive ability, do not know how, physical disabilities, not supplied by family, or no interest)	11 (31)		11 (32)		0	

^a^Missing responses from 2 assisted living facilities and 1 nursing home.

^b^Missing responses from 6 assisted living facilities.

### Changes in Facility ICTs, Access, and Use Since the Onset of the COVID-19 Pandemic

Since the onset of the COVID-19 pandemic and the subsequent lockdown of LTC facilities, 61% (43/70) of the LTC administrators reported an increase in technology spending at their facility (Table 4). A majority (37/70, 53%) of the LTC facilities reported purchasing ICTs for their residents. The main way the ICTs were purchased was by using facility funds (29/37, 78%). Though Centers for Medicare and Medicaid Services funding was provided for COVID-19 communicative technology grants for NHs, only 45% (5/11) of the nursing home administrators reported using this source of funding to purchase ICTs for their residents. ALF administrators also reported using personal funds, donations, and a small business loan. The top three ICTs purchased by LTC administrators for their residents (nonmutually exclusive) were tablet devices (27/37, 73%), smartphones (8/37, 22%), and laptops (8/37, 22%). In an open-ended question, 35 of the 37 administrators who purchased ICTs during the COVID-19 pandemic reported that the primary reason for purchasing ICTs was to help residents communicate with their family members. Additional reasons for purchasing ICTs included enabling telehealth and providing a secure communication channel for their staff.

**Table 4:** Changes in access to and use of information and communication technologies at facilities since the onset of the COVID-19 pandemic.

Changes			Total (N=70), n (%)	Assisted living facilities (n=58), n (%)	Nursing homes (n=12), n (%)
**Change in technology spending**					
	No change		27 (39)	27 (47)	0
	Increased spending less than 25%		20 (29)	16 (28)	4 (33)
	Increased spending by 25%-50%		15 (21)	10 (17)	5 (42)
	Increased spending by more than 50%		8 (11)	5 (9)	3 (25)
**Facility purchased information and communication technologies for residents’ use**					
	Yes		37 (53)	26 (45)	11 (92)
	No		33 (47)	32 (55)	1 (8)
**Among participants who reported purchasing information and communication technologies for residents’ use:**					
	**Funds used to purchase information and communication technologies**				
		Facility funds	29 (78)	21 (81)	8 (73)
		The Centers for Medicare and Medicaid Services COVID-19 communicative technology grant	5 (14)	0	5 (45)
		Donations	3 (8)	3 (12)	0
		Other (personal funds, small business loans, and residents provided for self)	4 (11)	4 (15)	0
	**Information and communication technologies purchased**				
		Tablet devices	27 (73)	16 (62)	11 (100)
		Smartphones	8 (22)	6 (23)	2 (18)
		Laptops	8 (22)	7 (27)	1 (9)
		Cellphones	1 (3)	1 (4)	0
		Other (Facebook portal, Amazon Echo, Nucleus, Eversound technology, headsets, cords to connect tablets and phones to televisions, and smart televisions)	8 (22)	8 (31)	0
	**How information and communication technologies have been used by residents^a^**				
		Videoconferencing with family members	31 (86)	21 (81)	10 (100)
		Videoconferencing with healthcare providers	26 (72)	19 (73)	7 (70)
		Videoconferencing with friends	25 (69)	16 (62)	9 (90)
		Playing games	10 (28)	8 (31)	2 (20)
		Shopping	9 (25)	6 (23)	3 (30)
		Searching for information	8 (22)	6 (23)	2 (20)
		Email	4 (11)	2 (8)	2 (20)
		Other (Pleasure, Telehealth)	3 (8)	3 (12)	0
	**How residents learned to use information and communication technologies^a^**				
		Staff-assisted	35 (97)	25 (96)	10 (100)
		Self-taught	6 (17)	3 (12)	3 (30)
		Other resident–assisted	4 (11)	3 (12)	1 (10)
		Other	1 (3)	0	1 (10)
		Do not know	1 (3)	1 (4)	0

^a^Missing response from one nursing home.

Administrators reported that, on average, 42% (SD 30.4%) of the residents used the technology provided by facilities and 25% (SD 26.4%; Table 5) of the residents were not able to use the technology provided by the facility owing to health or other impairments. Per the LTC administrators, residents have predominately used the newly purchased ICTs for videoconferencing with family members (31/36, 86%), health care providers (26/36, 72%), and friends (25/36, 69%). Residents have also used the ICTs for entertainment such as playing games (10/36, 28%), shopping (9/36, 25%), and searching for information (8/36, 22%). Though most of the LTC facilities did not have a dedicated person to assist residents with technology use, administrators reported that residents mainly learned to use the ICTs with help from LTC staff members (35/36, 97%).

**Table 5:** Additional changes in access to and use of information and communication technologies among facilities since the onset of the COVID-19 pandemic.

Additional changes	Residents (%), mean (SD); range
Used the technology provided by the facility	42.6 (30.4); 0-100
Unable to use the technology provided by the facility owing to health or other impairments	25.1 (26.4); 0-95

### Benefits of and Barriers to ICT Use

The most commonly reported benefits reported by LTC administrators were that using ICTs helped residents feel connected to their family members (26/34, 77%) and friends (16/34, 47%), and using ICTs allowed the residents to socialize more with others (11/34, 32%; Table 6). Administrators noted barriers to ICT use, such as staff not having time to assist residents with technology, broken technology, and residents who did not want to share technology, although these barriers were each reported by <25% (9/34) of respondents.

**Table 6:** Benefits of and Barriers to the use of information and communication technologies.

		Total (N=34), n (%)	Assisted living facilities (n=24), n (%)	Nursing homes (n=10), n (%)
**Benefits of using information and communication technologies**				
	Residents feel connected to family members	26 (77)	17 (71)	9 (90)
	Residents feel connected to friends	16 (47)	11 (46)	5 (50)
	Residents are socializing more	11 (32)	9 (38)	2 (20)
	Decreased negative behaviors from residents	7 (21)	6 (25)	1 (10)
	Residents feel connected to other residents	5 (15)	4 (17)	1 (10)
	Other (eased some anxiety for residents and family)	2 (6)	2 (8)	0
**Barriers to using information and communication technologies**				
	Staff do not have time to assist residents with technology	5 (15)	2 (8)	3 (30)
	Broken technology	4 (12)	2 (8)	2 (20)
	Residents do not want to share technology	2 (6)	1 (4)	1 (10)
	Other (not enough devices and staff to help with tech use and residents with dementia)	3 (9)	2 (8)	1 (10)

### Results of Binary Logistic Regression Analysis: Relationship Between ICT Purchase During the COVID-19 Pandemic and Facility Characteristics

Binary logistic regression analysis suggest that NHs, compared to ALFs, were 10.23 times more likely to purchase ICTs for residents’ use during the COVID-19 pandemic (odds ratio 11.23, 95% CI 1.12-113.02; *P*=.04). None of the other facility characteristics were related to whether LTC facilities purchased ICTs. The overall results of binary regression analysis for ICTs purchased during the COVID-19 pandemic are shown in Table 7.

**Table 7:** Results of binary logistic regression analysis for the relationship between the purchase of information and communication technologies and facility characteristics.

		Odds ratio (SE; 95% CI)	*P* value
Type (nursing home)		11.23 (1.18; 1.12-113.02)	.04
**Ownership**			
	For profit	1.85 (1.28; 0.15- 22.87)	.63
	Nonprofit	0.72 (1.39; 0.05-10.82)	.81
Bed size		1.00 (0.01; 0.99-1.01)	.68

## Discussion

### Principal Findings

This study is unique in that it presents an institutional perspective regarding how LTC facilities attempted to use ICTs to help address the socioemotional needs of their residents during the COVID-19 pandemic. Although there were some LTC facilities that, prior to the pandemic, provided ICTs for residents’ use, corroborating the findings from other prior studies [15,17,19], the advent of the lockdowns led many of the South Carolina facilities in this study to purchase ICTs.

NH administrators had higher odds of reporting that they purchased ICTs than ALF administrators. However, neither facility size nor ownership type were related to whether ICTs were purchased. Larger samples with more diversity in facility size, particularly among NHs, as well as other facility characteristics, might reveal differences that were obscured due to the homogeneity in NH respondent facility sizes in this study.

Since the onset of the COVID-19 pandemic, most of the LTC facilities in this study purchased ICTs, primarily tablet devices (27/37, 73%), to help enhance resident connection with social ties during the pandemic. Less than a quarter of the LTC facilities purchased smartphones or laptops for residents to use to communicate with friends, family, and health care providers during the lockdown. Although most of the LTC facilities did not have dedicated staff to assist residents in using ICTs, more than 95% (35/37) of the administrators in this study reported that staff helped residents learn to use ICTs during the pandemic to communicate with social ties and related reasons. This suggests that LTC facilities should consider having staff available to assist residents with using ICTs, thus confirming what other studies have suggested [16,18].

Though the LTC administrators in this study reported ICT use by their residents primarily for communication with their social ties, the majority (26/36; 72%) reported that residents used the ICTs for telehealth purposes. Given the high risk of COVID-19 among older adults, telehealth could be an important way for older adults to continue health care with minimal risks. While research is needed to explore how telehealth is used by LTC facilities and LTC residents in more detail, interventions are also needed to help older adults learn to use ICTs to effectively use in general and for telehealth services in particular [16,18].

### Strengths and Limitations

This is one of the few studies examining administrators’ technology adaptations during the COVID-19 pandemic. The results of this study illustrate the importance of staff members for helping residents to be able to use ICTs, as well as the fact that almost none of the facilities had a dedicated staff person to assist with technology needs at the time of this study. This suggests that facilities should take into account the technological needs of their residents and provide ongoing support to help them maintain their ICT use; prior research has noted the importance of ongoing technical support for older adults to be able to continue to use ICTs over time [16,18,20].

While this study sheds light on ICTs purchased and used in LTC facilities since the start of the pandemic, the data were collected from LTC facilities in South Carolina, which limits the generalizability of this study. Consistent with the LTC industry, our sample is predominately for profit LTC facilities. However, the majority bed size for both the ALFs and NHs in this study is not representative of the LTC facilities in South Carolina or the United States. Although 50% (29/58) of the ALFs in this study were medium-sized facilities (26-100 beds), the majority of ALFs in the United States (65%) and in South Carolina (46%) are small facilities (25 beds or less). In addition, 83% (10/12) of the NHs in this study were large facilities (101 beds or more), while the majority (64%) of NHs in the United States and in South Carolina (49%) are medium-sized facilities (26-100 beds) [63]. We acknowledge that the number of NHs that participated in the study was very small (n=12). Given the small number of NH administrators in the sample, the results for NHs should be taken with caution. It may be the case that a selection effect occurred with NHs who utilized ICTs in their facilities being more likely to respond to our ICT focused survey. Alternatively, perhaps larger NHs are more likely to have ICT access for their residents.

We found that prior to February 2020, there were NH administrators who reported that their residents used laptops (11/12, 92%), tablets (12/12, 100%), or smartphones (12/12, 100%). However, the number of NH administrators who reported residents having these was very small (n=12). Assisted living administrators also noted that prior to February 2020, their residents used laptops (27/58, 52%), tablet devices (38/58, 73%), and smartphones (49/58, 94%). Given the presence of greater health conditions among NH residents [64,65], compared to ALF residents, we would have expected that smaller percentages of NH residents would have been reported to use ICTs than what was reported in this study.

While the exploratory results of this study are informative in helping to illustrate the range of actions taken and administrators’ perceptions of these ICT use impacts on residents, additional data with larger and more diverse samples of LTC administrators as well as other staff members and residents are needed to ascertain if and how various types of LTC facilities adapted to the COVID-19 pandemic to help residents maintain connections to their social ties. Future research should investigate the types and degree of ICTs available for residents’ use in a national sample of LTC facilities, as well as identifying how LTC administrators adapted the ICTs available to LTC residents.

### Conclusions

LTC facilities were not adequately prepared to support the socioemotional needs of their residents in the event of a federally mandated facility lockdown [3]. ICT use can be a useful tool to help LTC residents maintain contact with social ties either during a pandemic or during nonpandemic times. However, LTC facilities and residents must have ICTs available to use, residents must be skilled in using ICTs, and support must be available to ensure continued use for residents to reap the benefits of their use. We encourage LTC facilities to develop technology integration plans to prepare for future situations that may affect LTC residents’ interaction and communication opportunities, such as another pandemic, and to facilitate residents’ use in the present time.

## Abbreviations

ALF: assisted living facility
DHEC: South Carolina Department of Health and Environmental Control
ICT: information and communication technology
LTC: long-term care
NH: nursing home
